# APOE ε4 Allele Dose and Time to Clinical Conversion from Mild Cognitive Impairment to Alzheimer’s Disease Dementia: An ADNI Survival Analysis

**DOI:** 10.3390/biomedicines14061280

**Published:** 2026-06-04

**Authors:** Faizaan Fazal Khan, Goo-Rak Kwon

**Affiliations:** Department of Information and Communication Engineering, Chosun University, Gwangju 61452, Republic of Korea; dkfaizaan12@gmail.com

**Keywords:** Alzheimer disease, mild cognitive impairment, apolipoprotein E4, disease progression, survival analysis, proportional hazards models, risk factors, neuroimaging

## Abstract

**Background/Objectives:** Existing Alzheimer’s disease (AD) prediction studies often treat APOE ε4 as a binary carrier variable and emphasize classification rather than time-to-event progression. This study evaluated whether APOE ε4 allele dose predicts clinical conversion from mild cognitive impairment (MCI) to AD dementia/probable AD in a longitudinal survival framework adjusted for hippocampal volume and baseline cognition. **Methods:** We analyzed 1115 Alzheimer’s Disease Neuroimaging Initiative (ADNI) participants with baseline MCI, APOE genotype data, and at least one follow-up visit, grouped by APOE ε4 allele count (0, 1, or 2). Kaplan–Meier curves, Bonferroni-corrected log-rank tests, nested Cox models, interaction testing, and twelve sensitivity and robustness analyses were performed. **Results:** During 3.73 ± 3.38 years of mean follow-up, 399 participants (35.8%) clinically converted. Median conversion-free survival was 18.47 years for non-carriers, 4.32 years for heterozygotes, and 3.41 years for homozygotes, although the non-carrier median occurred late in follow-up. In the fully adjusted Cox model, APOE ε4 dose remained associated with conversion hazard (HR = 1.580, 95% CI 1.362–1.834, *p* < 0.0001). Intracranial Volume (ICV)-adjusted hippocampal volume was protective (HR = 0.620, 95% CI 0.566–0.680, *p* < 0.0001), and the model achieved a Concordance Index (C-index) of 0.805. The APOE ε4 × hippocampal volume interaction was not significant (likelihood ratio test *p* = 0.098). Sensitivity analyses supported robustness, although the APOE ε4 association was attenuated in the exploratory amyloid-positive CSF subgroup. **Conclusions**: These findings support APOE ε4 allele dose as a statistical marker of clinical progression risk in ADNI, not as evidence of biomarker-confirmed AD progression or distinct mechanisms.

## 1. Introduction

Alzheimer’s disease (AD) is the most prevalent cause of dementia globally, with an estimated 55 million individuals currently affected and projections exceeding 139 million by 2050 [1]. Mild cognitive impairment (MCI) represents a clinically defined intermediate stage between normal aging and dementia, with annual conversion rates to AD ranging from 10% to 15% in community samples and considerably higher in specialty clinic cohorts [2]. Accurate prediction of which MCI patients will convert to AD, and how rapidly, carries direct implications for patient counselling, the design of clinical trials for disease-modifying therapies, and the timing of therapeutic interventions [3].

The apolipoprotein E epsilon 4 (APOE ε4) allele is the strongest known common genetic risk factor for late-onset AD. Heterozygous carriers face a two to threefold increase in lifetime risk, while homozygous carriers face a tenfold to fifteenfold increase relative to non-carriers [4]. Recent evidence from Fortea et al. (2024) has further suggested that APOE ε4 homozygosity may constitute a genetically distinct form of AD, characterized by near-complete penetrance of amyloid pathology by age 65 and an earlier onset of clinical symptoms [5]. Complementary work by Cai et al. (2025) demonstrated through meta-analysis and longitudinal validation that APOE ε4 acts as a conditional accelerator of hippocampal atrophy, with homozygotes declining over three times faster than non-carriers in the presence of amyloid positivity [6].

Despite this well-established association between APOE ε4 and AD risk, the specific relationship between allele dose and the timing of conversion from MCI to AD has received comparatively limited attention within a survival analysis framework. Prior prediction modelling studies have primarily focused on binary classification of converters versus non-converters using machine learning or logistic regression approaches [7,8]. Prior Magnetic Resonance Imaging (MRI)-based deep learning studies have achieved high diagnostic classification performance for distinguishing AD, MCI, and cognitively normal groups [9], but classification models generally do not directly estimate time to clinical conversion or provide survival probabilities. Chen et al. (2022) conducted a systematic review and meta-analysis of MCI-to-AD prediction models and identified substantial heterogeneity in predictive accuracy across studies, with most models lacking explicit time-to-event modelling [7]. Rye et al. (2022) similarly emphasized the need for clinically transferable predictive features rather than complex imaging-derived biomarkers [8].

Survival analysis methods, including Kaplan–Meier estimators and Cox proportional hazards regression, provide median time-to-conversion estimates and hazard ratios that are directly interpretable in clinical and trial planning contexts. Mirabnahrazam et al. (2023) applied deep survival analysis to predict time to conversion in ADNI using multimodal data, achieving strong discriminative performance but with limited interpretability of the genetic contribution [10]. Kim et al. (2024) similarly applied prognostic models to ADNI using functional connectome features, yet the independent contribution of APOE genotype dosage within a fully adjusted survival model remained underexplored [11].

Our recent ADNI-based longitudinal analysis further showed that APOE ε4 dose is associated with cognitive decline trajectories and may interact with hippocampal volume across the broader AD continuum [12]. However, that study focused on longitudinal cognitive trajectories rather than time-to-event conversion among participants with baseline MCI. This leaves a specific prognostic question unresolved: whether APOE ε4 allele dose predicts the timing of clinical conversion from MCI to AD dementia after accounting for baseline hippocampal volume and cognitive status.

At the same time, AD is increasingly conceptualized as a biologically defined disease rather than only as a clinical syndrome. The 2024 revised diagnostic criteria emphasize the role of core AD biomarkers in defining and staging AD, which is important when interpreting clinical MCI-to-dementia conversion studies [13]. Clinically diagnosed MCI is etiologically heterogeneous, and clinical AD dementia may include mixed or non-AD pathologies, including limbic-predominant age-related TDP-43 encephalopathy and primary age-related tauopathy [14,15]. Therefore, studies based on clinical diagnosis remain valuable for prognosis but should distinguish clinical conversion from biomarker-confirmed AD progression.

Although other genetic contributors to AD risk, including SORL1 and TREM2, are increasingly recognized, the present analysis focused on APOE ε4 allele dose because APOE genotype was available in the harmonized ADNI survival cohort and is directly interpretable in clinical and trial-enrichment contexts [16].

The present study builds on prior ADNI prognostic literature by applying dose-stratified Kaplan–Meier analysis and nested Cox proportional hazards regression to 1115 ADNI participants with baseline MCI, available APOE genotype data, and longitudinal clinical follow-up. Rather than presenting APOE ε4 as a newly identified risk factor, the study evaluates its incremental prognostic contribution as an ordinal allele-dose variable within a time-to-event framework adjusted for baseline hippocampal volume, cognitive status, and demographic covariates. The objectives were to estimate APOE ε4 group-specific clinical conversion trajectories, quantify the association between APOE ε4 allele count and time to clinical AD dementia diagnosis, assess whether this association persists after adjustment for structural and cognitive markers, and evaluate robustness through sensitivity analyses addressing missing data, hippocampal-source heterogeneity, MRI timing alignment, biomarker-defined subsamples, diagnostic reversion, and model assumptions.

## 2. Materials and Methods

### 2.1. Data Source and Study Population

Data were obtained from the Alzheimer’s Disease Neuroimaging Initiative (ADNI; adni.loni.usc.edu) [17] (last accessed: 17 April 2026), a multisite longitudinal study that collects standardized clinical, genetic, cognitive, and neuroimaging assessments across four sequential study phases (ADNI-1, ADNI-GO, ADNI-2, ADNI-3). The full ADNI master dataset used in this study comprised 15,530 longitudinal observations from 3761 unique participants, with observation dates spanning September 2005 to August 2025.

**Figure 1:**
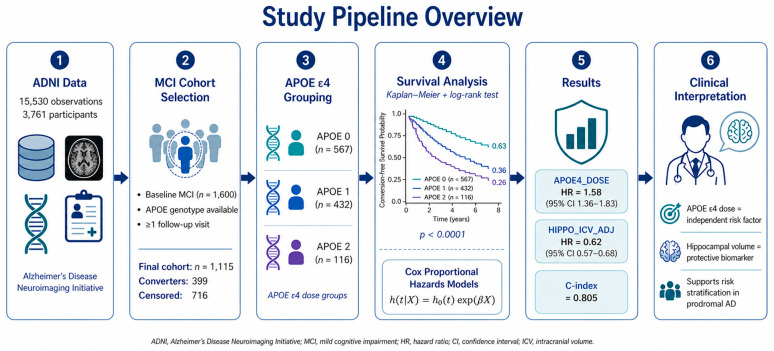
Study pipeline overview. ADNI data were filtered to a baseline MCI cohort (N = 1115) and analyzed using Kaplan–Meier and Cox models.

From the full dataset, participants were restricted to those with a baseline diagnosis of MCI (N = 1600). The analytic sample was further restricted to those meeting the following inclusion criteria: (a) available APOE ε4 genotype data, and (b) at least one follow-up visit beyond baseline (TIME_YEARS > 0). The application of these criteria yielded a final analytic sample of 1115 participants. The sample derivation process is summarized in Table 1, and an overview of the full data processing and survival analysis workflow is shown in Figure 1.

The pipeline includes cohort selection, APOE ε4 dose grouping, hippocampal volume harmonization, definition of time to AD conversion, and subsequent survival analyses using Kaplan–Meier estimation, Cox proportional hazards models, interaction testing, and sensitivity analyses. Additional cohort construction details are provided in Table S5.

### 2.2. Exposure Variables

The primary exposure was APOE ε4 allele dose, defined as the count of ε4 alleles (0, 1, or 2) derived by parsing the genotype string in the ADNI APOERES genetic database (e.g., a genotype of “3/4” yields dose = 1; a genotype of “4/4” yields dose = 2). Participants were stratified into three groups: non-carriers (dose 0, n = 567), heterozygotes (dose 1, n = 432), and homozygotes (dose 2, n = 116). APOE genotype is time-invariant; therefore, a single value per participant was merged onto all visits using the participant identifier (RID).

### 2.3. Outcome Variable

The primary outcome was clinical conversion from baseline MCI to AD dementia/probable AD, defined as an AD diagnosis recorded at any follow-up visit after baseline MCI. Amyloid or tau biomarker confirmation was not required for inclusion in the primary analysis; therefore, the outcome should be interpreted as clinical diagnostic progression rather than biomarker-confirmed progression from MCI due to AD to AD dementia. The event indicator was coded as Event = 1 for converters and Event = 0 for participants who remained clinically diagnosed as MCI or reverted to cognitively normal status without subsequent AD diagnosis during follow-up. For converters, event time was defined as the first follow-up visit at which AD was recorded. For censored participants, event time was defined as the last observed follow-up visit. Time was computed as the difference in days between each visit date and the participant’s earliest recorded visit date, divided by 365.25.

### 2.4. Covariates

Baseline values for all covariates were extracted as the first valid non-missing observation per participant from the longitudinal master dataset. The following covariates were included.

ICV-adjusted hippocampal volume (HIPPO_ICV_ADJ) was derived as the ratio of total bilateral hippocampal volume to intracranial volume (ICV), scaled by a factor of 1000 to yield values in a clinically interpretable range. Hippocampal volume was sourced preferentially from the UCD White Matter Hyperintensity (WMH) file, where TOTAL_BRAIN and TOTAL_CSF were used to estimate ICV, and supplemented for participants not covered by this source with values from the University of California, San Francisco (UCSF) ADNI FreeSurfer cross-sectional version 7 pipeline (UCSFFSX7; ADNI file: All_Subjects_UCSFFSX7_26Aug2025.csv, accessed on 17 April 2026). Because the UCD WMH file uses visit coding that could not be aligned one-to-one with the monthly ADNI diagnostic visit codes in the merged dataset, hippocampal data were merged on participant identifier only, using the earliest available scan per participant as the baseline hippocampal value. A total of 3168 participants across the full database had hippocampal data available; within the analytic MCI sample of 1115, only 5 participants (0.45%) had missing hippocampal volume and were imputed to the sample median (3.287). Outliers more than 4 standard deviations from the mean were screened, and none were identified.

Because hippocampal volume was harmonized from UCD_WMH and UCSFFSX7 imaging-derived sources, we evaluated temporal alignment between the hippocampal MRI scan date and the baseline clinical diagnosis date when both dates were available. The absolute difference between MRI and baseline clinical assessment dates was summarized in days and months. A sensitivity analysis was then conducted by restricting the sample to participants whose hippocampal MRI occurred within ±6 months of baseline clinical assessment.

Additional covariates included Mini-Mental State Examination score (MMSCORE, valid range 0 to 30), Clinical Dementia Rating Sum of Boxes (CDRSB), age at baseline (AGE, years), sex (SEX, coded 0 = male, 1 = female), and years of education (PTEDUCAT). Within the analytic sample, missingness for these covariates was extremely low: MMSCORE and CDRSB each had 1 missing value (0.09%), and SEX, AGE, and PTEDUCAT had no missing values. All missing covariate values were imputed to the sample median for continuous variables and the sample mode for categorical variables prior to Cox model fitting.

Geriatric Depression Scale (GDS) scores were summarized descriptively but were not included in the primary Cox model because depressive symptoms were not part of the primary genetic, structural MRI, cognitive, and demographic predictor set. GDS had higher missingness in the cleaned master dataset than the primary model covariates, although missingness in the analytic MCI cohort was modest at 23/1115 participants (2.06%). To address the possibility that excluding GDS affected the APOE ε4 estimate, we fitted an additional GDS-adjusted sensitivity model using the same median-imputation approach.

### 2.5. Statistical Methods

#### 2.5.1. Kaplan–Meier Analysis

Kaplan–Meier survival functions were estimated separately for each APOE ε4 dose group. The survival function S(t) represents the probability of remaining in MCI status at time t, estimated as:

(1) $$S\left(t\right)=\prod_{t_{i}\leq t}\left(1-\frac{d_{i}}{n_{i}}\right)$$

where $n_{i}$ is the number at risk, and $d_{i}$ is the number of events at time $t_{i}$. The overall difference across groups was tested using the multivariate log-rank statistic. Pairwise comparisons between all three dose groups were conducted using the log-rank test with Bonferroni correction, yielding an adjusted significance threshold of α = 0.0167 for three simultaneous comparisons. Median survival time was defined as the time at which S(t) first falls to or below 0.50. Survival probabilities were extracted at landmark timepoints of 2, 4, 6, 8, and 10 years. Ninety-five percent Wilson confidence intervals were used for observed conversion rates, as these have superior coverage properties to Wald intervals when proportions are not near 0.50.

#### 2.5.2. Cox Proportional Hazards Regression

Cox proportional hazards models were fitted to estimate hazard ratios (HR) for conversion risk associated with each covariate. The Cox model specifies the hazard function as:

(2) $$h\left(t|X\right)=h_{0}\left(t\right)\exp\left(\beta_{1}X_{1}+\beta_{2}X_{2}+\cdots +\beta_{p}X_{p}\right)$$

where h_0_(t) is the unspecified baseline hazard, and X_1_ through X_p_ are covariates. Three nested models were fitted sequentially, as described in Table 2.

Model discrimination was assessed using the concordance index (C-index), defined as the proportion of concordant pairs among all evaluable subject pairs. Higher C-index values indicate better discriminative ability, with 0.5 representing chance and 1.0 representing perfect discrimination. Model fit was assessed using the Akaike Information Criterion (AIC), with lower AIC indicating better fit. Multicollinearity among primary model covariates was evaluated using variance inflation factors calculated from standardized predictors. The proportional hazards (PH) assumption was evaluated for each covariate in Model 3 using Schoenfeld residuals, implemented via the check_assumptions function in the lifelines Python library.

To evaluate whether an ordinal APOE ε4 allele-dose specification was appropriate, we compared the primary ordinal APOE4_DOSE model with a categorical two-indicator specification using dose 0 as the reference group and separate indicators for dose 1 and dose 2. Model fit was compared using AIC, and the categorical contrasts were used to assess whether the carrier groups showed a strictly monotonic pattern.

#### 2.5.3. Interaction Analysis

An interaction term (APOE4_DOSE × HIPPO_ICV_ADJ) was created as the product of the two variables and added to the primary model covariates. The interaction model was compared to the primary model (Model 3) using a likelihood ratio test (LRT), with the test statistic distributed as χ^2^(1) under the null hypothesis of no interaction. A significant LRT indicates that the effect of APOE ε4 dose on conversion risk differs by level of hippocampal volume.

#### 2.5.4. Sensitivity Analyses

Twelve sensitivity and robustness analyses were conducted to assess the stability of the primary APOE ε4 dose finding, as described in Table 3. The first set of analyses evaluated whether the result was affected by homozygote group size, minimal covariate imputation, depressive symptoms, short follow-up, and hippocampal-volume operationalization. A second set addressed hippocampal-source heterogeneity by adding HIPPO_SOURCE as a covariate, restricting the analysis to UCD_WMH-derived hippocampal measurements, and replacing HIPPO_ICV_ADJ with a source-wise hippocampal z-score. Additional reviewer-motivated analyses evaluated MRI timing alignment, amyloid-positive CSF status, MCI-to-CN diagnostic reversion, and proportional hazards robustness.

Because hippocampal volume was harmonized from UCD_WMH and UCSFFSX7 sources, S6–S8 assessed potential source-related measurement heterogeneity. S6 added HIPPO_SOURCE as a covariate to the primary Cox model, S7 restricted the analysis to participants with UCD_WMH-derived hippocampal measurements, and S8 replaced HIPPO_ICV_ADJ with a hippocampal z-score standardized within each imaging source. S9 evaluated temporal alignment by restricting the sample to participants whose hippocampal MRI occurred within ±6 months of baseline. S10 was an exploratory analysis restricted to amyloid-positive participants with available baseline CSF data. S11 excluded participants who reverted from MCI to cognitively normal status without subsequent AD diagnosis. S12 evaluated whether the APOE ε4 estimate remained stable after addressing potential proportional hazards concerns for AGE and CDRSB using time-varying and stratified Cox specifications.

#### 2.5.5. Software

All analyses were performed in Python version 3.10.20 on Ubuntu 24.04 LTS, using Visual Studio Code version 1.111.0 as the integrated development environment. Data processing and statistical analyses used pandas version 2.1.4, NumPy version 1.26.3, SciPy version 1.12.0, statsmodels version 0.14.1, lifelines version 0.28.0, scikit-learn version 1.4.0, pingouin version 0.5.4, openpyxl version 3.1.2, XlsxWriter version 3.1.9, and nbformat version 5.9.2. Figures were generated using matplotlib version 3.8.2 and seaborn version 0.13.1. Analyses were conducted in Jupyter version 1.0.0 notebooks. Statistical significance was set at alpha = 0.05 (two-sided) unless otherwise specified.

## 3. Results

### 3.1. Baseline Characteristics

The analytic sample comprised 1115 MCI participants with a mean follow-up of 3.73 ± 3.38 years (range 0.04 to 18.52 years) and a median follow-up of 2.99 years; this figure represents time to first AD diagnosis for converters and time to last observed visit for censored participants, consistent with standard right-censored survival data. Of these, 399 participants (35.8%) clinically converted to AD dementia/probable AD during follow-up, and 716 (64.2%) were censored. The APOE ε4 dose distribution was 567 non-carriers (50.9%), 432 heterozygotes (38.7%), and 116 homozygotes (10.4%). Baseline characteristics stratified by dose group are summarized in Table 4.

As shown in Table 4, non-carriers were the oldest group at baseline (77.03 ± 8.32 years) and homozygotes the youngest (72.76 ± 7.11 years), consistent with earlier clinical presentation in ε4 carriers. ADAS-Cog (Alzheimer’s Disease Assessment Scale, Cognitive Subscale) increased monotonically with dose (15.03, 17.61, 18.02; *p* < 0.0001), indicating progressively worse baseline cognitive performance in carriers. Mini-Mental State Examination (MMSE) was highest in non-carriers (27.73) and lowest in heterozygotes (26.95), with homozygotes intermediate (27.14); MMSE differences were significant across groups (*p* < 0.0001), though the absolute differences were modest. Hippocampal volume showed a monotonic decline across dose groups (2.98, 2.75, 2.55; *p* = 0.0002), consistent with the established dose-dependent effect of APOE ε4 on medial temporal lobe atrophy [6]. Clinical Dementia Rating, Sum of Boxes (CDR-SB) was highest in heterozygotes (1.84 ± 1.37) rather than homozygotes (1.62 ± 0.90), though the homozygote versus heterozygote difference was not significant after Bonferroni correction (*p* = 0.109). Sex, education, and depression scores did not differ across dose groups. Follow-up duration was significantly shorter in the higher dose groups (4.27, 3.24, 2.90 years; *p* < 0.0001), reflecting faster conversion and earlier exit from follow-up in ε4 carriers.

Bonferroni-corrected pairwise comparisons for hippocampal volume confirmed significant differences between dose 0 and dose 1 (*p* = 0.009) and between dose 0 and dose 2 (*p* = 0.001), but not between dose 1 and dose 2 (*p* = 0.301). The same threshold pattern was observed for MMSE and ADAS-Cog, where dose 0 differed significantly from both carrier groups, but dose 1 and dose 2 did not differ from each other, foreshadowing the conversion trajectory results reported below.

### 3.2. Missing Data

**Figure 2:**
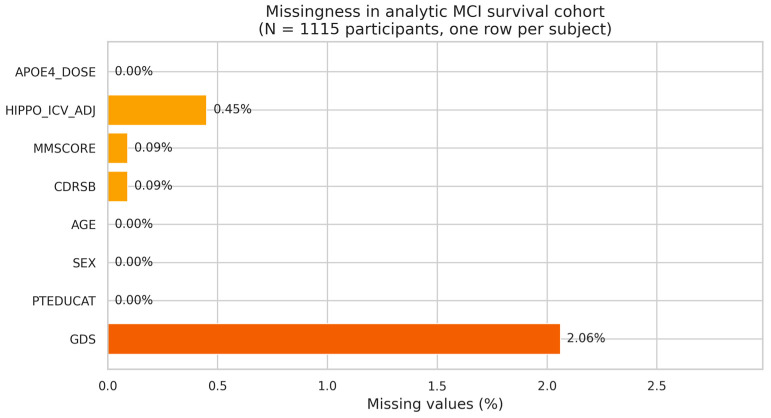
Missingness in the analytic MCI survival cohort. Bars show the percentage of missing values before imputation among 1115 participants. The darker orange bar highlights GDS, which had the highest missingness; other colored bars indicate primary Cox model covariates with non-zero missingness.

Within the analytic MCI survival cohort, missingness was negligible for all primary Cox model covariates: HIPPO_ICV_ADJ was missing for 5 participants (0.45%), MMSCORE for 1 participant (0.09%), CDRSB for 1 participant (0.09%), and APOE4_DOSE, AGE, SEX, and PTEDUCAT had no missing values. GDS had 23 missing values (2.06%) and was included only in the GDS-adjusted sensitivity model. The missingness pattern in the analytic cohort is summarized in Figure 2. Detailed variable definitions and missingness handling are provided in Table S1.

### 3.3. Kaplan–Meier Survival Analysis

**Figure 3:**
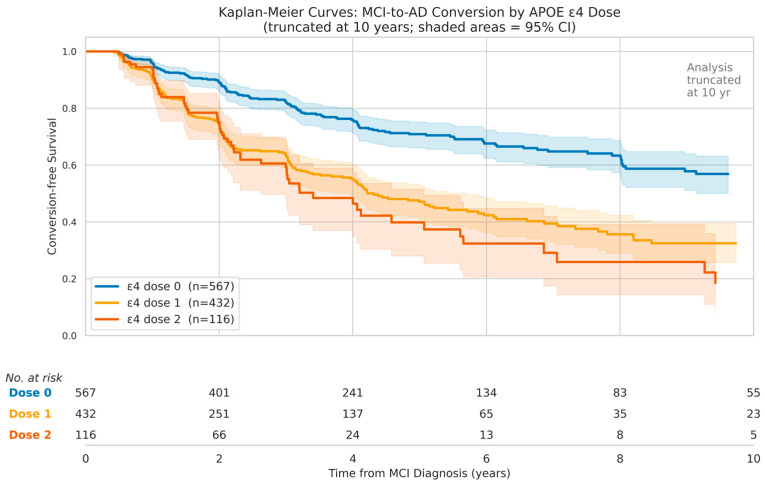
Kaplan–Meier survival curves for clinical MCI-to-AD dementia conversion by APOE ε4 dose group. Curves are truncated at 10 years to avoid overinterpretation of sparse late follow-up. Shaded regions indicate 95% confidence intervals, and the risk table shows the number of participants remaining under observation at each timepoint.

Kaplan–Meier survival curves stratified by APOE ε4 dose group are presented in Figure 3. The overall multivariate log-rank test was highly significant (chi-squared = 71.16, *p* < 0.0001), confirming significant differences in conversion-free survival across dose groups. Non-carriers showed consistently higher survival probabilities than both carrier groups across follow-up, while the difference between heterozygotes and homozygotes was smaller and not statistically significant in pairwise testing.

Median time to conversion was 18.47 years for non-carriers, 4.32 years for heterozygotes, and 3.41 years for homozygotes. The gap in median conversion-free time between non-carriers and heterozygotes was 14.15 years, and between non-carriers and homozygotes was 15.06 years. Pairwise log-rank tests with Bonferroni correction confirmed significant differences between non-carriers and heterozygotes (*p* < 0.0001) and between non-carriers and homozygotes (*p* < 0.0001). Heterozygotes and homozygotes did not differ significantly from each other (*p* = 0.163). The pairwise results are presented in Table 5.

As shown in Table 5, the strongest survival separation occurred between non-carriers and ε4 carriers. The absence of a significant pairwise difference between heterozygotes and homozygotes indicates that Kaplan–Meier separation was more clearly carrier-dominant than strictly linear across all three dose groups. Therefore, the survival results should be interpreted as showing shorter conversion-free survival among ε4 carriers, with limited additional KM separation between the two carrier groups.

Landmark survival probabilities and restricted mean survival time provide more stable summaries within the observed follow-up window. Landmark survival probabilities at clinically relevant timepoints are summarized in Table 6. At 4 years, 75.7% of non-carriers remained unconverted compared with 55.2% of heterozygotes and 46.4% of homozygotes. By 10 years, 56.9% of non-carriers remained conversion-free, compared with 32.5% of heterozygotes and 18.5% of homozygotes. At 10 years, the number at risk was 55 for dose 0, 23 for dose 1, and 5 for dose 2. RMST through 10 years was 7.41 years for dose 0, 5.39 years for dose 1, and 4.75 years for dose 2, supporting shorter conversion-free time among ε4 carriers while avoiding overinterpretation of the late Kaplan–Meier tail.

**Figure 4:**
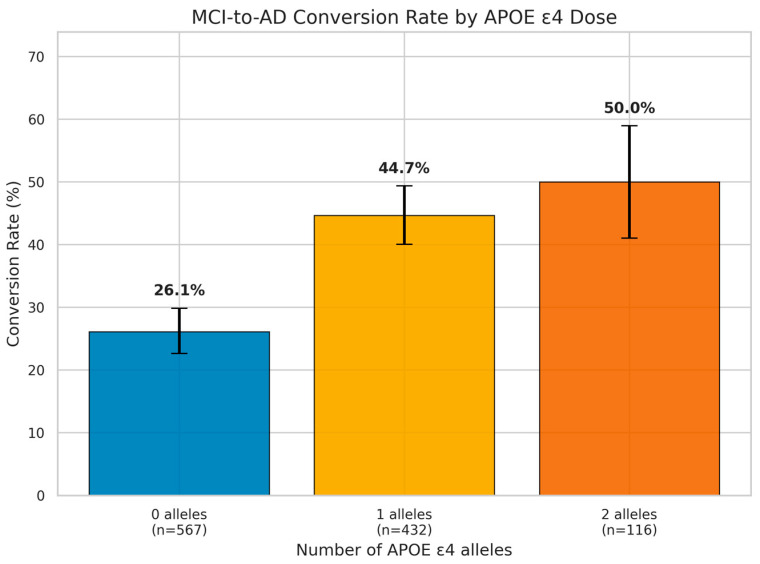
Observed clinical MCI-to-AD dementia conversion rates by APOE ε4 dose group with 95% Wilson confidence intervals.

As shown in Table 6, non-carriers maintained a survival probability above 0.50 through 10 years of follow-up (S(10 yr) = 0.569), while heterozygotes crossed the 0.50 threshold near 4.32 years and homozygotes near 3.41 years. At 2 years, survival probabilities were similar across carrier groups (0.741 vs. 0.751 for doses 1 and 2), consistent with the early crossover visible in Figure 3. The cumulative conversion rates with Wilson confidence intervals are shown in Figure 4.

**Figure 5:**
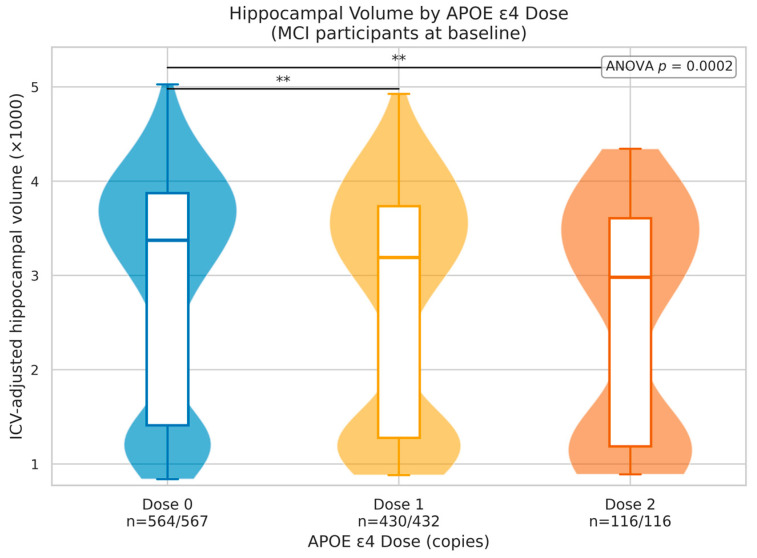
Distribution of ICV-adjusted hippocampal volume by APOE ε4 dose group. Violin plots show group-level distributions, and embedded boxplots show the median and interquartile range. Group-specific sample sizes are shown below each dose category. The omnibus ANOVA was significant (*p* = 0.0002). Horizontal brackets indicate Bonferroni-corrected pairwise comparisons. ** indicates significant differences between dose 0 and dose 1 (*p* = 0.009) and between dose 0 and dose 2 (*p* = 0.001).

The hippocampal volume distribution by APOE ε4 dose group is shown in Figure 5. Each distribution represents one participant-level baseline hippocampal measurement from the analytic survival cohort rather than longitudinal visit-level observations. HIPPO_ICV_ADJ was calculated as bilateral hippocampal volume divided by intracranial volume and scaled by 1000. Non-missing hippocampal measurements were available for 564/567 non-carriers, 430/432 heterozygotes, and 116/116 homozygotes. The omnibus ANOVA was significant (F = 8.637, *p* = 0.0002), indicating group-level differences in ICV-adjusted hippocampal volume. Bonferroni-corrected pairwise comparisons showed significantly lower hippocampal volume in dose 1 compared with dose 0 (*p* = 0.009) and in dose 2 compared with dose 0 (*p* = 0.001), while dose 1 and dose 2 did not differ significantly from each other (*p* = 0.301). Thus, the hippocampal distribution supports lower baseline hippocampal volume among ε4 carriers, with the clearest separation occurring between non-carriers and carriers. Potential source-related measurement heterogeneity was further evaluated through source-adjusted, UCD_WMH-only, and source-wise z-score sensitivity analyses.

### 3.4. Cox Model Results

Before fitting the nested models, we compared an ordinal APOE4_DOSE specification with a categorical two-indicator specification to evaluate whether treating APOE ε4 allele count as an ordinal predictor was reasonable. The ordinal specification showed slightly better model fit than the categorical specification (AIC = 4556.19 vs. 4558.09) and was retained for parsimony and interpretability. However, because the AIC difference was small and Kaplan–Meier pairwise comparisons showed stronger separation between non-carriers and carriers than between heterozygotes and homozygotes, the Cox results should be interpreted as evidence of increasing conversion hazard across APOE ε4 dose groups rather than definitive proof of a strict additive biological dose-response. Results of the three nested models are summarized in Table 7. In the unadjusted model (Model 1), each additional APOE ε4 allele was associated with a 75.4% increase in conversion hazard (HR = 1.754, 95% CI 1.531 to 2.009, *p* < 0.0001), with a C-index of 0.608. After partial adjustment for hippocampal volume, MMSE, and CDR-SB in Model 2, the hazard ratio attenuated modestly to 1.599 (95% CI 1.382 to 1.848, *p* < 0.0001), and the C-index improved substantially to 0.803, reflecting the strong discriminative contribution of the cognitive and structural covariates. In the fully adjusted primary model (Model 3), the APOE ε4 dose hazard ratio was 1.580 (95% CI 1.362 to 1.834, *p* < 0.0001), with a C-index of 0.805.

As shown in Table 7, the APOE ε4 dose hazard ratio remained statistically significant across all three models. The attenuation from Model 1 (HR = 1.754) to Model 3 (HR = 1.580) indicates partial overlap between APOE ε4-associated risk and baseline structural or cognitive disease severity. However, the persistence of the APOE ε4 association after adjustment should be interpreted as a residual statistical association, not as evidence of a distinct biological pathway. The near-identical C-index values for Models 2 and 3 indicate that age, sex, and education added little discrimination beyond the cognitive and structural covariates. The C-index increase, from 0.608 in Model 1 to 0.805 in Model 3, was driven primarily by the addition of cognitive and structural covariates in Model 2, rather than by APOE ε4 alone.

**Figure 6:**
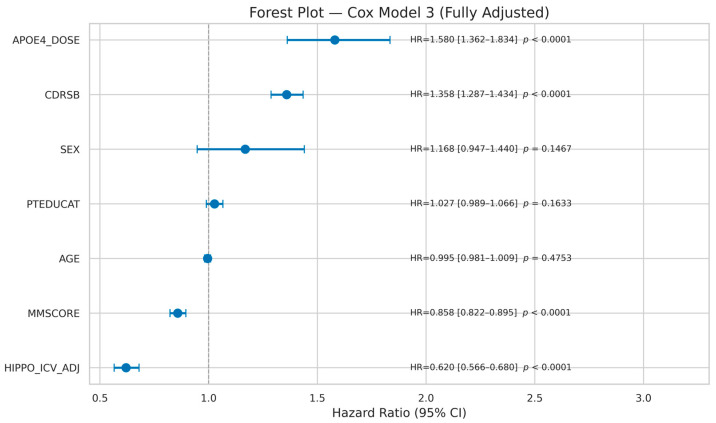
Forest plot of hazard ratios from the fully adjusted Cox proportional hazards model. Points represent hazard ratios, horizontal bars represent 95% confidence intervals, and the dashed vertical line indicates HR = 1.0.

The full set of predictor hazard ratios from the primary model (Model 3) is presented in Table 8 and displayed graphically in Figure 6.

As shown in Table 8, APOE ε4 dose, hippocampal volume, MMSE, and CDR-SB were statistically significant predictors of conversion in the fully adjusted model. APOE4_DOSE (HR = 1.580) and HIPPO_ICV_ADJ (HR = 0.620) both retained significant associations after mutual adjustment, suggesting that genotype and baseline hippocampal volume each contributed prognostic information within the adjusted statistical model. This should not be interpreted as evidence of separate biological pathways. CDR-SB had the largest effect per unit among the cognitive measures (HR = 1.358 per point), while MMSE was protective (HR = 0.858 per point). Age, sex, and education did not reach statistical significance. The forest plot for Model 3 is presented in Figure 6.

Variance inflation factors were low for all primary model predictors: APOE4_DOSE = 1.060, HIPPO_ICV_ADJ = 1.223, MMSCORE = 1.311, CDRSB = 1.190, AGE = 1.087, SEX = 1.052, and PTEDUCAT = 1.050. These values were all well below commonly used thresholds for problematic multicollinearity, indicating that collinearity among the primary Cox model covariates was unlikely to affect the model estimates. Pairwise Pearson correlation coefficients among the primary Cox model covariates are shown in Figure S1 and further support the absence of substantial multicollinearity.

### 3.5. Proportional Hazards Assumption

Updated Schoenfeld residual testing in the final analytic cohort indicated that the primary predictor APOE4_DOSE satisfied the proportional hazards assumption on the rank-transformed test (*p* = 0.175). HIPPO_ICV_ADJ (*p* = 0.268), MMSCORE (*p* = 0.913), CDRSB (*p* = 0.172), SEX (*p* = 0.290), and PTEDUCAT (*p* = 0.854) also satisfied the proportional hazards assumption. AGE showed borderline evidence of non-proportionality but did not reach the conventional 0.05 significance threshold (*p* = 0.087). To evaluate whether this borderline pattern affected the APOE ε4 estimate, we fitted additional robustness models allowing time-varying effects for AGE and CDRSB and a stratified Cox model using AGE and CDRSB categories. APOE ε4 remained statistically significant in both robustness models: HR = 1.471, *p* < 0.0001 in the time-varying model and HR = 1.575, *p* < 0.0001 in the stratified Cox model. These results support the stability of the primary APOE ε4 association despite borderline age-related non-proportionality. Detailed proportional hazards diagnostic results are provided in Table S2.

### 3.6. APOE ε4 × Hippocampal Volume Interaction Results 

The likelihood ratio test comparing the interaction model (APOE4_DOSE × HIPPO_ICV_ADJ added to Model 3) with the primary model yielded an LRT statistic of 2.73 with 1 degree of freedom (*p* = 0.098). The interaction was not statistically significant at the conventional alpha = 0.05 threshold. This indicates that we did not observe statistical evidence of multiplicative effect modification between APOE ε4 dose and baseline hippocampal volume in this cohort. However, the absence of a statistically significant interaction should not be interpreted as evidence that no biological interaction exists, particularly given the smaller homozygote subgroup. APOE ε4 dose and hippocampal volume were therefore interpreted as jointly informative baseline predictors in the adjusted statistical model, rather than as evidence of independent biological mechanisms.

### 3.7. Sensitivity and Robustness Results 

Twelve sensitivity and robustness analyses assessed the stability of the APOE ε4 dose association from the primary Cox model. These analyses evaluated the influence of homozygote group size, minimal covariate imputation, GDS adjustment, short follow-up, hippocampal-volume operationalization, hippocampal-source heterogeneity, MRI timing alignment, amyloid-positive CSF status, MCI-to-CN diagnostic reversion, and proportional hazards robustness. Results are summarized in Table 9. Additional source-specific sensitivity analyses are reported in Table S3, and a complete summary of all sensitivity and robustness analyses is provided in Table S4.

As shown in Table 9, the APOE ε4 dose association remained statistically significant in nearly all sensitivity and robustness analyses. In the GDS-adjusted model, the APOE ε4 estimate was essentially unchanged relative to the primary model (HR = 1.585, 95% CI 1.365–1.840, *p* < 0.0001), and GDS itself was not independently associated with conversion in the adjusted model (HR = 1.039, *p* = 0.128), indicating that adjustment for depressive symptoms did not materially affect the primary association. Among participants with available baseline CSF data, 443 met amyloid-positive criteria. In this exploratory amyloid-positive subgroup, the APOE ε4 estimate was attenuated and did not reach conventional statistical significance (HR = 1.179, 95% CI 0.982–1.416, *p* = 0.0768; C-index = 0.7786), suggesting that part of the APOE ε4 association in the full clinically defined MCI cohort may reflect enrichment for underlying amyloid pathology among ε4 carriers. MRI timing alignment also supported robustness: 682/732 participants with available date information had hippocampal MRI within ±6 months of baseline, and the APOE ε4 association remained significant in this restricted subgroup (HR = 1.403, 95% CI 1.189–1.655, *p* < 0.0001; C-index = 0.8299). A total of 104 censored participants reverted from MCI to cognitively normal status without subsequent AD diagnosis, including 69 non-carriers, 33 heterozygotes, and 2 homozygotes. Excluding these reverters yielded a consistent APOE ε4 association (HR = 1.393, 95% CI 1.222–1.587, *p* < 0.0001; C-index = 0.7886). In proportional-hazards robustness analyses, APOE ε4 remained significant in both the time-varying AGE/CDRSB model (HR = 1.471, 95% CI 1.271–1.703, *p* < 0.0001) and the stratified AGE/CDRSB model (HR = 1.575, 95% CI 1.364–1.820, *p* < 0.0001). Overall, these analyses support the robustness of the primary APOE ε4 association while clarifying that the estimate is attenuated in the amyloid-positive CSF subgroup.

## 4. Discussion

This study characterized APOE ε4 dose-stratified clinical MCI-to-AD dementia conversion trajectories in 1115 ADNI participants over a mean follow-up of 3.73 years. The principal finding is that APOE ε4 allele count remained strongly associated with clinical conversion risk after adjustment for baseline hippocampal volume, cognitive status, and demographic variables. Each additional ε4 allele was associated with a 58% increase in conversion hazard in the fully adjusted Cox model (HR = 1.580, *p* < 0.0001). Kaplan–Meier analysis showed shorter conversion-free survival among ε4 carriers, but the non-carrier median occurred late in follow-up when the risk set was smaller; therefore, landmark survival probabilities and RMST provide more stable clinical summaries.

An important methodological concern was the harmonization of hippocampal volume across UCD_WMH and UCSFFSX7 imaging sources. This concern was directly addressed through three additional sensitivity analyses. The APOE ε4 effect remained significant after adjustment for HIPPO_SOURCE, when restricted to UCD_WMH-derived hippocampal measurements only, and after source-wise hippocampal z-score standardization. These findings indicate that the primary APOE ε4 survival association is not driven by hippocampal source heterogeneity.

### 4.1. APOE ε4 as an Adjusted Statistical Predictor

A central distinction between the present analysis and smaller prior ADNI MCI analyses is that APOE ε4 dose remained statistically robust after adjustment for hippocampal volume and cognitive measures. The modest attenuation of the hazard ratio from the unadjusted model (HR = 1.754) to the fully adjusted model (HR = 1.580) indicates partial overlap between APOE ε4-associated risk and baseline neurodegenerative or cognitive severity. However, this adjusted association should not be interpreted as evidence of a separate biological pathway [4,5].

The statistical significance of both APOE4_DOSE (HR = 1.580) and HIPPO_ICV_ADJ (HR = 0.620) in the same fully adjusted model suggests that genotype and baseline hippocampal volume each contributed prognostic information within the adjusted model. This is consistent with the established understanding that APOE ε4 is associated with amyloid accumulation and downstream hippocampal neurodegeneration [6]. However, APOE genotype and hippocampal volume likely reflect different timescales of the pathological cascade, with genotype representing lifelong susceptibility and hippocampal volume reflecting accumulated structural injury at baseline. These adjusted associations should not be interpreted as evidence of distinct biological pathways.

### 4.2. Carrier-Dominant Separation with Increasing APOE ε4-Associated Risk

Kaplan–Meier and Cox analyses together suggest that the strongest survival separation occurs between non-carriers and ε4 carriers. Pairwise log-rank tests showed significant differences between non-carriers and both carrier groups, whereas heterozygotes and homozygotes did not differ significantly in Kaplan–Meier comparison. The categorical Cox specification showed higher risk for both carrier groups compared with non-carriers and a higher point estimate for homozygotes than heterozygotes. However, the survival curves overlapped between the two carrier groups, and the homozygote group was smaller. Therefore, the findings are best interpreted as a robust carrier-related association with increasing APOE ε4-associated risk, rather than proof of a strict additive biological dose response.

### 4.3. Non-Significant Interaction Between APOE ε4 Dose and Hippocampal Volume

The likelihood ratio test for the APOE4_DOSE × HIPPO_ICV_ADJ interaction was not statistically significant (LRT *p* = 0.098), suggesting no clear evidence of multiplicative effect modification between genetic and structural risk markers. This finding differs from a preliminary, smaller-sample analysis in which interaction effects were less stable. The current analysis includes 399 conversion events and therefore provides a more reliable estimate. Clinically, the result suggests that APOE ε4 genotype and hippocampal volume can be interpreted as complementary predictors of conversion risk, rather than requiring a hippocampal-volume-specific APOE effect.

### 4.4. Comparison with Prior Survival Analyses

The median conversion times observed in this study, 18.47 years for non-carriers versus approximately 4 years for ε4 carriers, are substantially longer for non-carriers than those reported in smaller prior cohort studies. This reflects the composition of the full ADNI MCI population across four study phases, which includes participants with a wide range of baseline clinical severity and many participants who are followed for only a few years before being censored. The 35.8% overall conversion rate is lower than in earlier ADNI phases because later ADNI phases enrolled participants at earlier and milder stages of MCI. This is appropriate for a study examining the full range of MCI-to-AD conversion trajectories rather than a high-conversion enriched sample. The C-index of 0.805 is comparable to the range reported by Mirabnahrazam et al. (2023) for multimodal deep survival models (0.73 to 0.82 depending on modality combination) [10], despite our model using only clinically available variables without advanced imaging-derived features.

### 4.5. Limitations

Several limitations warrant acknowledgment. First, conversion was defined using clinical AD diagnosis rather than amyloid or tau biomarker confirmation. MCI is a heterogeneous clinical syndrome, and clinically diagnosed AD dementia may include mixed or non-AD pathologies such as limbic-predominant age-related TDP-43 encephalopathy or primary age-related tauopathy. Conversely, some censored participants may have had biological AD without clinical conversion during follow-up. The exploratory amyloid-positive CSF sensitivity analysis showed attenuation of the APOE ε4 estimate, supporting the view that part of the APOE ε4 association in the full cohort may reflect enrichment for underlying AD pathology among ε4 carriers. Therefore, the present findings should be interpreted as predictors of clinical diagnostic progression in ADNI rather than as definitive estimates of biomarker-confirmed progression from MCI due to AD to AD dementia.

Second, the persistence of the APOE ε4 association after adjustment for hippocampal volume and cognitive measures should not be interpreted as evidence of separate biological pathways. Hippocampal atrophy, MMSE, and CDR-SB may partly lie downstream of APOE-related amyloid, tau, or neurodegenerative processes. The adjusted APOE estimate, therefore, reflects residual statistical association after accounting for baseline structural and clinical severity, not a formal mediation result.

Third, non-informative censoring was assumed. Follow-up duration differed by APOE ε4 group, and although shorter follow-up among carriers is partly explained by earlier conversion, differential dropout, mortality, or study attrition cannot be fully excluded because death dates and competing-event indicators were unavailable. Fine–Gray and true cause-specific competing-risk models could therefore not be fitted.

Fourth, ADNI is a highly selected research cohort with relatively high education, specialty-clinic recruitment, and limited population diversity. The reported hazard ratios, survival trajectories, and RMST estimates may not generalize directly to more diverse or community-based MCI populations. External validation in independent cohorts is required before clinical implementation.

Fifth, the analysis used baseline predictors only. ADNI includes longitudinal cognitive, imaging, and biomarker trajectories, but the present study was designed to evaluate baseline prognostic associations. Future work should evaluate time-varying cognition, longitudinal hippocampal atrophy, amyloid/tau biomarkers, broader genetic predictors, including SORL1 and TREM2, and external validation cohorts.

Sixth, several secondary analyses, including the amyloid-positive CSF subgroup, MRI timing restriction, MCI-to-CN reversion exclusion, and proportional hazards robustness models, should be interpreted as sensitivity or exploratory analyses rather than confirmatory hypothesis tests. Apart from Bonferroni correction for pairwise Kaplan–Meier comparisons, no formal multiplicity correction was applied across all secondary analyses.

Seventh, ADNI spans multiple phases with evolving recruitment procedures, diagnostic practices, and biomarker availability. Although ADNI provides standardized clinical data, phase-related diagnostic and protocol heterogeneity may have influenced estimated conversion rates.

Eighth, APOE ε4 dose groups differed at baseline in age, cognition, hippocampal volume, and follow-up duration, which may reflect differences in disease stage at enrollment as well as possible survivor or selection effects, particularly because non-carriers were older at baseline. Finally, MMSE and CDR-SB overlap conceptually with clinical diagnostic domains used to define progression, so their inclusion as predictors may introduce some criterion contamination. These issues do not invalidate the APOE ε4 association but limit causal and individual-level interpretation.

### 4.6. Clinical Implications

These findings may inform research-level risk stratification and trial-enrichment strategies, but they should not be interpreted as sufficient for direct individual-level clinical prognostication without calibration, external validation, biomarker confirmation, and decision-curve evaluation. APOE ε4 genotyping at the point of MCI diagnosis may provide a simple and interpretable research-level stratification variable for identifying groups at elevated risk of earlier clinical conversion. Although the estimated median conversion-free survival was much longer for non-carriers than for ε4 carriers, the late timing of the non-carrier median means that landmark probabilities may be more clinically useful for counselling and trial planning. At 4 years, 75.7% of non-carriers remained unconverted compared with 55.2% of heterozygotes and 46.4% of homozygotes; by 10 years, the corresponding probabilities were 56.9%, 32.5%, and 18.5%. These estimates may support genotype-stratified follow-up planning and trial enrichment in research settings, pending external validation and calibration. The adjusted associations of APOE ε4 dose and hippocampal volume support further evaluation of combined genetic and neuroimaging markers for research-level risk stratification.

## 5. Conclusions

APOE ε4 allele dose was strongly associated with time to clinical conversion from MCI to AD dementia/probable AD in ADNI. Each additional ε4 allele was associated with a 58% increase in conversion hazard in the fully adjusted Cox model (HR = 1.580, 95% CI 1.362 to 1.834, *p* < 0.0001), after adjustment for baseline hippocampal volume, MMSE, CDR-SB, age, sex, and education. ICV-adjusted hippocampal volume, MMSE, and CDR-SB were also statistically significant predictors, indicating that genetic, structural, and cognitive markers provide complementary statistical information in the adjusted model. Kaplan–Meier analysis showed the strongest survival separation between non-carriers and ε4 carriers, while heterozygotes and homozygotes did not differ significantly in pairwise comparison. The APOE ε4 dose by hippocampal volume interaction was not statistically significant (LRT *p* = 0.098), although this does not exclude modest biological interaction. The APOE ε4 association remained robust across most sensitivity and robustness analyses, including source-adjusted, UCD_WMH-only, source-wise hippocampal z-score, MRI timing, diagnostic reversion, GDS-adjusted, and proportional hazards robustness models, but was attenuated in the exploratory amyloid-positive CSF subgroup. These findings support APOE ε4 allele dose as a clinically interpretable statistical marker of progression risk in ADNI, while emphasizing the need for biomarker confirmation, calibration, and external validation before individual-level clinical application.

## Figures and Tables

**Table 1:** Sample derivation from the ADNI MCI cohort.

Criterion	N
Total participants in ADNI master dataset	3761
Baseline MCI diagnosis	1600
Excluded: missing APOE ε4 genotype	455
Excluded: no follow-up visit beyond baseline	30
Final analytic sample	1115

**Table 2:** Specification of nested Cox proportional hazards models.

Model	Covariates Included	Rationale
Model 1 (unadjusted)	APOE4_DOSE	Crude genetic effect
Model 2 (partial)	APOE4_DOSE, HIPPO_ICV_ADJ, MMSCORE, CDRSB	Adjustment for structural and cognitive biomarkers
Model 3 (primary)	APOE4_DOSE, HIPPO_ICV_ADJ, MMSCORE, CDRSB, AGE, SEX, PTEDUCAT	Full adjustment including demographic confounders

**Table 3:** Sensitivity and robustness analysis specifications.

Label	Description	Rationale
S1	Exclude APOE ε4 homozygotes	Tests whether the result depends on the smallest dose group
S2	Complete cases only, no imputation	Assesses effect of minimal covariate imputation
S3	Add GDS to the primary Cox model	Tests whether depressive symptoms affect the APOE ε4 estimate
S4	Restrict to participants with at least 2 follow-up visits	Excludes participants with minimal observation time
S5	Hippocampal volume as binary median-split variable	Tests hippocampal operationalization
S6	Add HIPPO_SOURCE to the primary Cox model	Tests imaging-source heterogeneity
S7	Restrict to UCD_WMH-derived hippocampal values only	Tests robustness within primary hippocampal source
S8	Replace HIPPO_ICV_ADJ with source-wise hippocampal z-score	Removes source-scale differences
S9	Restrict to MRI within ±6 months of baseline	Tests temporal alignment of hippocampal MRI
S10	Restrict to amyloid-positive CSF subgroup	Exploratory amyloid-positive CSF subgroup analysis
S11	Exclude MCI-to-CN reverters	Tests effect of diagnostic reversion
S12	PH-robust model with time-varying or stratified AGE/CDRSB handling	Tests robustness to proportional hazards diagnostics

**Table 4:** Baseline characteristics by APOE ε4 dose group (N = 1115).

Variable	Dose 0 (n = 567)	Dose 1 (n = 432)	Dose 2 (n = 116)	Test	*p*-Value
Age, years	77.03 ± 8.32	75.72 ± 7.35	72.76 ± 7.11	ANOVA	<0.0001
Education, years	16.07 ± 2.71	15.84 ± 2.82	16.06 ± 2.71	ANOVA	0.391
MMSE	27.73 ± 2.15	26.95 ± 2.53	27.14 ± 2.29	ANOVA	<0.0001
CDR-SB	1.53 ± 1.20	1.84 ± 1.37	1.62 ± 0.90	ANOVA	0.0004
ADAS-Cog	15.03 ± 6.57	17.61 ± 6.94	18.02 ± 6.26	ANOVA	<0.0001
HIPPO_ICV_ADJ	2.98 ± 1.17	2.75 ± 1.22	2.55 ± 1.18	ANOVA	0.0002
GDS	1.90 ± 1.80	1.89 ± 1.87	1.79 ± 1.44	ANOVA	0.825
Follow-up, years	4.27 ± 3.68	3.24 ± 2.98	2.90 ± 2.72	ANOVA	<0.0001
Female, N (%)	237 (41.8%)	177 (41.0%)	51 (44.0%)	Chi-square	0.843
Converted, N (%)	148 (26.1%)	193 (44.7%)	58 (50.0%)	Chi-square	<0.0001

Note: Continuous variables are reported as mean ± Standard Deviation (SD) and tested by one-way Analysis of Variance (ANOVA). Categorical variables are reported as N (%) and tested by chi-square. HIPPO_ICV_ADJ = ICV-adjusted hippocampal volume scaled by ×1000.

**Table 5:** Pairwise log-rank test results with Bonferroni correction (adjusted alpha = 0.0167).

Comparison	N (A)	N (B)	Log-Rank Statistic	*p*-Value	Significant
Dose 0 vs. Dose 1	567	432	54.06	<0.0001	Yes
Dose 0 vs. Dose 2	567	116	46.74	<0.0001	Yes
Dose 1 vs. Dose 2	432	116	1.95	0.163	No

**Table 6:** Kaplan–Meier survival summary by APOE ε4 dose group.

Statistic	Dose 0 (n = 567)	Dose 1 (n = 432)	Dose 2 (n = 116)
Events (converters)	148	193	58
Conversion rate (%)	26.1 [22.7, 29.9]	44.7 [40.1, 49.4]	50.0 [41.0, 59.0]
Median survival (years)	18.47	4.32	3.41
S(2 yr)	0.890	0.741	0.751
S(4 yr)	0.757	0.552	0.464
S(6 yr)	0.676	0.423	0.324
S(8 yr)	0.633	0.356	0.259
S(10 yr)	0.569	0.325	0.185
RMST 0–10 yr	7.41	5.39	4.75

Note: Conversion rates are shown with 95% Wilson confidence intervals in brackets. S(t) = Kaplan–Meier estimated probability of remaining MCI-stable at year t.

**Table 7:** APOE ε4 dose hazard ratios across three nested Cox proportional hazards models (N = 1115, 399 events).

Model	HR (per Allele)	95% CI	*p*-Value	C-Index	AIC
Model 1 (unadjusted)	1.754	1.531 to 2.009	<0.0001	0.608	4942.54
Model 2 (partial)	1.599	1.382 to 1.848	<0.0001	0.803	4554.71
Model 3 (primary)	1.580	1.362 to 1.834	<0.0001	0.805	4556.19

**Table 8:** Full predictor hazard ratios from the primary Cox model (Model 3, N = 1115, 399 events).

Predictor	HR	95% CI	*p*-Value	Interpretation
APOE4_DOSE	1.580	1.362 to 1.834	<0.0001	Higher ε4 dose was associated with higher conversion hazard in the ordinal Cox model
HIPPO_ICV_ADJ	0.620	0.566 to 0.680	<0.0001	Each unit increase reduces hazard by 38%
MMSCORE	0.858	0.822 to 0.895	<0.0001	Each additional MMSE point reduces hazard by 14%
CDRSB	1.358	1.287 to 1.434	<0.0001	Each additional CDR-SB point increases hazard by 36%
AGE	0.995	0.981 to 1.009	0.475	Not independently significant
SEX	1.168	0.947 to 1.440	0.147	Not independently significant
PTEDUCAT	1.027	0.989 to 1.066	0.163	Not independently significant

**Table 9:** Sensitivity and robustness analyses for the APOE ε4 dose association.

Analysis	N	Events	APOE ε4 HR	95% CI	*p*-Value	C-Index
Primary reference	1115	399	1.580	1.362–1.834	<0.0001	0.8047
S1: Exclude homozygotes	999	341	1.625	1.299–2.034	<0.0001	0.8083
S2: Complete cases only	1108	398	1.579	1.361–1.833	<0.0001	0.8046
S3: GDS-adjusted	1115	399	1.585	1.365–1.840	<0.0001	0.8049
S4: Minimum 2 follow-up visits	1001	394	1.597	1.376–1.855	<0.0001	0.8010
S5: Hippocampal binary median split	1115	399	1.558	1.345–1.806	<0.0001	0.8050
S6: Source-adjusted	1108	398	1.449	1.272–1.651	<0.0001	0.8080
S7: UCD_WMH only	768	206	1.489	1.252–1.770	<0.0001	0.8102
S8: Source z-score	1108	398	1.447	1.271–1.647	<0.0001	0.8043
S9: MRI within ±6 months	682	258	1.403	1.189–1.655	<0.0001	0.8299
S10: Amyloid-positive CSF subgroup	443	226	1.179	0.982–1.416	0.0768	0.7786
S11: Exclude MCI-to-CN reverters	1011	399	1.393	1.222–1.587	<0.0001	0.7886
S12a: Time-varying AGE/CDRSB	1115	399	1.471	1.271–1.703	<0.0001	Not directly comparable
S12b: Stratified AGE/CDRSB	1115	399	1.575	1.364–1.820	<0.0001	Not directly comparable

Note: HR = hazard ratio; CI = confidence interval; PH = proportional hazards; C-index = concordance index. S12a and S12b were fitted to evaluate robustness to potential proportional hazards concerns for AGE and CDRSB. Their C-index values should not be interpreted as direct improvements over the primary model because the model structures differ from the primary Cox specification.

## Data Availability

The data analyzed in this study are available from the Alzheimer’s Disease Neuroimaging Initiative (ADNI) database. Because ADNI data are governed by data-use agreements, the authors do not have permission to redistribute the raw data directly. The data are publicly accessible to qualified researchers upon request and approval through the ADNI website (adni.loni.usc.edu). Analysis code and preprocessing scripts are available to the editor and reviewers upon reasonable request during peer review and will be made publicly available after acceptance through the following GitHub repository: https://github.com/FaizaanFazal/apoe4-mci-ad-survival-analysis.git, accessed on 30 May 2026.

## Supplementary Materials

The following supporting information can be downloaded at: https://www.mdpi.com/article/10.3390/biomedicines14061280/s1, Table S1: Missing data summary for the cleaned ADNI master dataset and analytic MCI survival cohort; Table S2: Proportional hazards assumption testing for the fully adjusted Cox model; Table S3: Hippocampal-source sensitivity analyses; Table S4: Full sensitivity analysis summary for APOE ε4 dose; Table S5: ADNI cohort construction and analytic sample derivation. Figure S1. Pearson correlation matrix of primary Cox model covariates. Values represent pairwise Pearson correlation coefficients among covariates included in the fully adjusted Cox model.

## Abbreviations

The following abbreviations are used in this manuscript:

AD	Alzheimer’s Disease
ADAS-Cog	Alzheimer’s Disease Assessment Scale, Cognitive Subscale
ADNI	Alzheimer’s Disease Neuroimaging Initiative
AIC	Akaike Information Criterion
ANOVA	Analysis of Variance
APOE	Apolipoprotein E
CDR-SB	Clinical Dementia Rating, Sum of Boxes
CI	Confidence Interval
C-index	Concordance Index
FreeSurfer	Freesurfer Image Analysis Suite
GDS	Geriatric Depression Scale
HR	Hazard Ratio
ICV	Intracranial Volume
LRT	Likelihood Ratio Test
MCI	Mild Cognitive Impairment
MMSE	Mini-Mental State Examination
MRI	Magnetic Resonance Imaging
PH	Proportional Hazards
SD	Standard Deviation
UCSF	University of California, San Francisco
