# Supplementary material for: APOE ε4 Allele Dose and Time to Clinical Conversion from Mild Cognitive Impairment to Alzheimer’s Disease Dementia: An ADNI Survival Analysis
Source: Biomedicines. 2026 Jun 4;14(6):1280. doi: 10.3390/biomedicines14061280 (PMC13297606; doi:10.3390/biomedicines14061280)
Supplement: Supplementary file 1 [file biomedicines-14-01280-s001.zip › biomedicines-4327896-supplementary.pdf]

Supplementary tables:

Supplementary Table S1 Missing data summary for the cleaned ADNI master dataset and analytic MCI survival cohort.

Variable	Missing in cleaned master dataset, n/N (%)	Missing in analytic MCI survival cohort, n/N (%)	Handling in analysis
APOE4_DOSE	1,665/15,530 (10.7%)	0/1,115 (0.0%)	Required for inclusion
HIPPO_ICV_ADJ	721/15,530 (4.6%)	5/1,115 (0.45%)	Median imputation
MMSCORE	3,182/15,530 (20.5%)	1/1,115 (0.09%)	Median imputation
CDRSB	3,278/15,530 (21.1%)	1/1,115 (0.09%)	Median imputation
ADAS_COG	3,213/15,530 (20.7%)	Not used in Cox model	Descriptive only
AGE	13/15,530 (0.1%)	0/1,115 (0.0%)	Complete
SEX	13/15,530 (0.1%)	0/1,115 (0.0%)	Complete
PTEDUCAT	13/15,530 (0.1%)	0/1,115 (0.0%)	Complete
GDS	4,316/15,530 (27.8%)	23/1,115 (2.06%)	Descriptive; included in GDS-adjusted sensitivity model

Note: The analytic MCI survival cohort included participants with baseline MCI, available APOE genotype data, and at least one follow-up visit. ADAS_COG and GDS were used descriptively and were not included in the primary Cox proportional hazards model. GDS was additionally evaluated in a GDS-adjusted sensitivity model.

Supplementary Table S2 Proportional hazards assumption testing for the fully adjusted Cox model.

Covariate	Rank-transformed Schoenfeld test p-value	Interpretation
APOE4_DOSE	0.175	PH assumption acceptable
HIPPO_ICV_ADJ	0.268	PH assumption acceptable
MMSCORE	0.913	PH assumption acceptable
CDRSB	0.172	PH assumption acceptable in the final model
AGE	0.087	Borderline but not significant at α = 0.05
SEX	0.290	PH assumption acceptable
PTEDUCAT	0.854	PH assumption acceptable

Note: PH = proportional hazards. APOE4_DOSE satisfied the proportional hazards assumption in the final fully adjusted model. AGE showed borderline evidence of non-proportionality but did not reach the conventional $\alpha = 0.05$ threshold. Additional time-varying and stratified Cox robustness models were fitted to evaluate whether potential AGE/CDRSB non-proportionality affected the APOE ε4 estimate.

Supplementary Table S3 Hippocampal-source sensitivity analyses.

Sensitivity analysis	Model description	N	Events	APOE4_DOSE HR	95% CI	p-value	C-index	Interpretation
S6: Source-adjusted	Primary Cox model plus HIPPO_SOURCE covariate	1,108	398	1.449	1.272 to 1.651	<0.0001	0.8080	APOE4 association remained significant after adjusting for hippocampal source
S7: UCD_WMH only	Primary Cox model restricted to UCD_WMH-derived hippocampal values	768	206	1.489	1.252 to 1.770	<0.0001	0.8102	APOE4 association remained significant within the primary hippocampal source
S8: Source z-score	HIPPO_ICV_ADJ replaced with source-wise standardized hippocampal z-score	1,108	398	1.447	1.271 to 1.647	<0.0001	0.8043	APOE4 association remained significant after removing source-scale differences

Note: These analyses tested whether the primary APOE ε4 association was affected by harmonizing hippocampal volume from UCD_WMH and UCSFFSX7 sources. The APOE4_DOSE effect remained statistically significant across all source-related sensitivity analyses.

Supplementary Table S4 Full sensitivity and robustness analysis summary for APOE ε4 dose.

Analysis	N	Events	APOE4_DOSE HR	95% CI	p-value	C-index
Primary reference model	1,115	399	1.580	1.362 to 1.834	<0.0001	0.8047
S1: Exclude homozygotes	999	341	1.625	1.299 to 2.034	<0.0001	0.8083
S2: Complete cases only	1,108	398	1.579	1.361 to 1.833	<0.0001	0.8046

Analysis	N	Events	APOE4_DOSE HR	95% CI	p-value	C-index
S3: GDS-adjusted[1]	1,115	399	1.585	1.365 to 1.840	<0.0001	0.8049
S4: Minimum 2 follow-up visits	1,001	394	1.597	1.376 to 1.855	<0.0001	0.8010
S5: Hippocampal binary median split	1,115	399	1.558	1.345 to 1.806	<0.0001	0.8050
S6: Source-adjusted	1,108	398	1.449	1.272 to 1.651	<0.0001	0.8080
S7: UCD_WMH only	768	206	1.489	1.252 to 1.770	<0.0001	0.8102
S8: Source z-score	1,108	398	1.447	1.271 to 1.647	<0.0001	0.8043
S9: MRI within ±6 months	682	258	1.403	1.189 to 1.655	<0.0001	0.8299
S10: Amyloid-positive CSF subgroup	443	226	1.179	0.982 to 1.416	0.0768	0.7786
S11: Exclude MCI-to-CN reverters	1,011	399	1.393	1.222 to 1.587	<0.0001	0.7886
S12a: Time-varying AGE/CDRSB	1,115	399	1.471	1.271 to 1.703	<0.0001	Not directly comparable
S12b: Stratified AGE/CDRSB	1,115	399	1.575	1.364 to 1.820	<0.0001	Not directly comparable

Note: The APOE4_DOSE hazard ratio remained positive and statistically significant across most sensitivity and robustness specifications, supporting the stability of the primary association. The exploratory amyloid-positive CSF subgroup showed attenuation of the APOE ε4 estimate and did not reach conventional statistical significance. S12a and S12b were fitted to evaluate robustness to proportional hazards concerns for AGE and CDRSB; their C-index values are not directly comparable with the primary Cox model because the model structures differ. The GDS-adjusted model (S3) yielded an AIC of 4555.99 versus 4556.19 for the primary model; the negligible improvement of 0.20 AIC units reflects the absence of independent prognostic contribution from GDS (HR = 1.039, p = 0.128).

[1] In the GDS-adjusted model, GDS was not independently associated with conversion risk (HR = 1.039, p = 0.128).

Supplementary Table S5 ADNI cohort construction and analytic sample derivation.

Step	Criterion	N remaining	Excluded at step
1	Total participants in cleaned ADNI master dataset	3,761	—
2	Baseline MCI diagnosis	1,600	2,161
3	Available APOE ε4 genotype	1,145	455
4	At least one follow-up visit beyond baseline	1,115	30
Final	Analytic MCI survival cohort	1,115	—

Note: The final analytic cohort consisted of baseline MCI participants with APOE genotype data and at least one post-baseline follow-up visit. Conversion time was defined as time to first AD diagnosis for converters and time to last available follow-up for censored participants.

Supplementary figures:

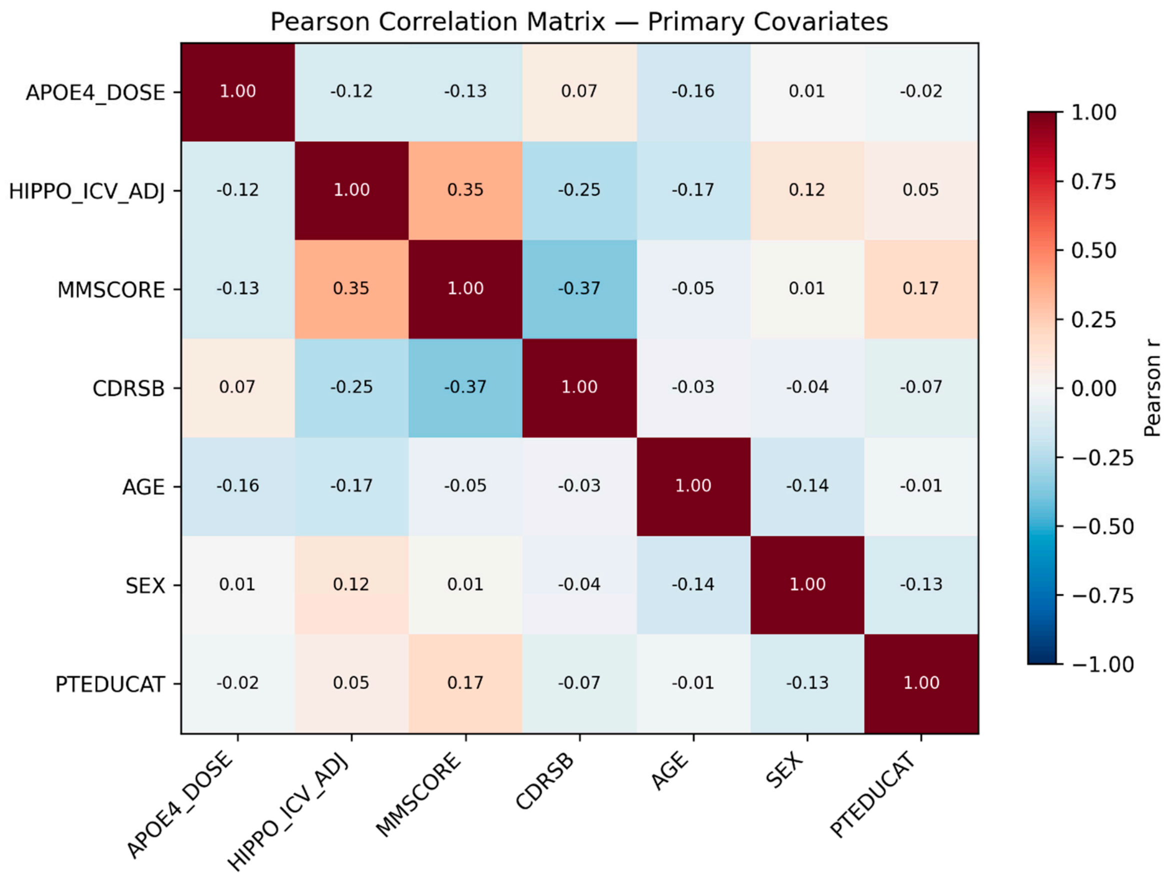

Supplementary Figure S1 Pearson correlation matrix of primary Cox model covariates. Values represent pairwise Pearson correlation coefficients among covariates included in the fully adjusted Cox model.
